# Navigational Signals for Insect and Slug Parasitic Nematodes: The Role of Ascorbate–Glutathione System and Volatiles Released by Insect-Damaged Sweet Pepper Roots

**DOI:** 10.3390/insects15100805

**Published:** 2024-10-15

**Authors:** Žiga Laznik, Mitja Križman, Jure Zekič, Mihaela Roškarič, Stanislav Trdan, Andreja Urbanek Krajnc

**Affiliations:** 1 Department of Agronomy, Biotechnical Faculty, University of Ljubljana, Jamnikarjeva 101, SI-1000 Ljubljana, Slovenia; stanislav.trdan@bf.uni-lj.si; 2 National Institute of Chemistry, Hajdrihova 19, SI-1001 Ljubljana, Sloveniajure.zekic@ki.si (J.Z.); 3 Faculty of Agriculture and Life Sciences, University of Maribor, Pivola 10, SI-2311 Hoče, Sloveniaandreja.urbanek@um.si (A.U.K.)

**Keywords:** *Capsicum annuum*, *Agriotes lineatus*, ascorbate–glutathione system, root volatile compounds, entomopathogenic nematodes, slug parasitic nematodes

## Abstract

**Simple Summary:**

This study explores how a wireworm (*Agriotes lineatus* L. [Coleoptera: Elateridae]) infestation affects sweet pepper (*Capsicum annuum* L.) plants and their interactions with parasitic nematodes (Nematoda: Rhabditidae). We found that *A. lineatus* damage decreases ascorbate levels in leaves but increases them in roots, along with higher cysteine and glutathione levels in leaves. These changes likely boost the plant’s antioxidant defense mechanisms. We also observed increases in carotenoids and chlorophylls, which directly enhance the plant’s protection against light stress. Additionally, wireworm-infested roots released several volatile compounds, among which, most notably, is hexanal that signals plant stress. Nematodes responded differently to tested exudates based on their species and environmental factors like temperature. The findings emphasize the need to consider these variables when using VOCs for the biological control of nematodes. Future research should focus on refining these strategies for better pest management in agriculture.

**Abstract:**

This study of underground multitrophic communication, involving plant roots, insects, and parasitic nematodes, is an emerging field with significant implications for understanding plant–insect–nematode interactions. Our research investigated the impact of wireworm (*Agriotes lineatus* L. [Coleoptera: Elateridae]) infestations on the ascorbate–glutathione system in sweet pepper (*Capsicum annuum* L.) plants in order to study the potential role in root-exudate-mediated nematode chemotaxis. We observed that an *A. lineatus* infestation led to a decrease in leaf ascorbate levels and an increase in root ascorbate, with corresponding increases in the glutathione content in both roots and leaves. Additionally, a pigment analysis revealed increased carotenoid and chlorophyll levels and a shift towards a de-epoxidized state in the xanthophyll cycle. These changes suggest an individual and integrated regulatory function of photosynthetic pigments accompanied with redox modifications of the ascorbate–glutathione system that enhance plant defense. We also noted changes in the root volatile organic compound (VOC). Limonene, methyl salicylate, and benzyl salicylate decreased, whereas hexanal, neoisopulegol, nonanal, phenylethyl alcohol, m-di-tert-butylbenzene, and trans-β-ionone increased in the roots of attacked plants compared to the control group. Most notably, the VOC hexanal and amino acid exudate cysteine were tested for the chemotaxis assay. Nematode responses to chemoattractants were found to be species-specific, influenced by environmental conditions such as temperature. This study highlights the complexity of nematode chemotaxis and suggests that VOC-based biological control strategies must consider nematode foraging strategies and environmental factors. Future research should further explore these dynamics to optimize nematode management in agricultural systems.

## 1. Introduction

Plants have developed various defenses against herbivores, including the emission of volatile organic compounds (VOCs) from their roots when attacked by insects. These VOCs play a crucial role in the interaction between plants, herbivores, and their predators [1,2,3,4]. They act as semiochemicals, affecting both herbivores and their enemies. Different VOCs are released in response to mechanical damage or specific herbivore feeding, influencing soil organisms by attracting, repelling, or poisoning them [1,5,6].

To assess volatile organic compounds (VOCs) for plant protection, modern gas chromatography-mass spectrometry (GC-MS) is essential. This technique uniquely identifies compounds at trace levels [1,2,3]. Given the complex nature of plant tissues and soils and the low concentration of VOCs, precise sampling techniques are necessary to enrich and extract target analytes for accurate analysis [1].

In response to biotic stress, plants often show systemic reactions. Soil-dwelling herbivores that damage roots can significantly alter metabolites in the plant’s aboveground parts [7,8,9,10]. Antioxidants and enzymes, particularly through the ascorbate–glutathione cycle, play a key role in managing oxidative stress and activating defense mechanisms. This cycle regulates redox signaling and boosts plant defenses [8]. While this system’s role in aboveground defense is well-documented [9,10], its function in belowground stress responses is less understood [8]. Root damage from herbivores increases jasmonate (JA) production [10], which enhances antioxidant defenses and stimulates glutathione synthesis [11]. Research shows that both glutathione and ascorbate are secreted from roots into the rhizosphere in response to stress [12,13,14,15].

Understanding how antioxidant defense pathways mediate communication between aboveground and belowground plant parts is still incomplete, especially regarding their spatial and temporal dynamics. This process is influenced by photosynthetic carbon fixation and photorespiration, which produce glycine for glutathione (GSH) synthesis [16]. Biotic stress can cause chlorophyll degradation and alter the chlorophyll a/b ratio [17]. Additionally, the xanthophyll cycle’s de-epoxidation state, linked to light energy dissipation, indicates the activation level and reactive oxygen species (ROS)’s production [17].

Nematodes are widespread across diverse ecosystems and habitats [18]. They include both free-living and parasitic species, with some offering benefits by controlling agricultural and forestry pests. Beneficial nematodes, like entomopathogenic nematodes (EPNs), are soft-bodied roundworms that parasitize insects [18]. They use chemical cues such as carbon dioxide and VOCs to find their hosts [1,2,5]. Notable families used in pest management are Heterorhabditidae and Steinernematidae [18]. The genus *Phasmarhabditis* Andrassy includes species that target pest gastropods [19], with 16 known species globally [20], while *Oscheius*, part of the Rhabditidae family, consists of free-living nematodes that feed on bacteria [21].

Early research focused mainly on aboveground plant communication due to methodological limitations [22]. Recently, however, there has been growing interest in underground multitrophic interactions involving plant roots, herbivores, and parasitic nematodes. Rasmann et al. [1] demonstrated that damaged maize roots release volatile organic compounds (VOCs) that affect the behavior of entomopathogenic nematodes (EPNs) like *Heterorhabditis megidis* Poinar, Jackson, and Klein. This finding has sparked a new field of study in underground multitrophic communication, supported by several publications [2,5]. While research on EPN responses to root-emitted VOCs is still limited, existing studies show that injured roots release specific VOCs, mostly investigated in maize, but also in other plants [2,3,5].

Species such as *Steinernema feltiae* Filipjev, *Steinernema carpocapsae* [Weiser], *Heterorhabditis bacteriophora* Poinar, *Phasmarhabditis papillosa* Schneider Andrássy, and *Oscheius myriophilus* [Poinar] play crucial roles in biological pest control, offering environmentally friendly alternatives to chemical pesticides [21,23,24]. *Steinernema* and *Heterorhabditis* species are widely used as EPNs, which effectively parasitize and kill a range of insect pests, including soil-dwelling larvae and other agricultural pests [21]. *S. feltiae* and *S. carpocapsae* are particularly important for controlling the larvae of flies, moths, and beetles, while *H. bacteriophora* is used to combat white grubs and other damaging soil pests [21].

On the other hand, *P. papillosa* and *O. myriophilus* have significant applications in mollusc management, specifically targeting harmful slug species [23,24]. These nematodes parasitize slugs, offering a biological method to reduce damage to crops caused by these gastropods. The use of these species in integrated pest management (IPM) supports sustainable agriculture, minimizing the need for harmful chemical inputs while preserving the ecosystem balance and enhancing biodiversity.

This study aimed to investigate the chemical communication between parasitic nematodes (*S. feltiae*, *S. carpocapsae*, *H. bacteriophora*, *P. papillosa*, and *O. myriophilus*) and both damaged and undamaged sweet pepper roots (*Capsicum annuum* L.). Wireworms (*Agriotes lineatus* L.; Coleoptera, Elateridae) were used to create root damage. The research had four goals: (1) to understand how wireworm attacks affect the ascorbate–glutathione system and photosynthetic pigments in plants; (2) to expand knowledge on tritrophic interactions among nematodes, insects, and plants; (3) to analyze changes in organic compound emissions from pepper roots due to herbivory; and (4) to determine if these root exudates influence nematode behavior.

## 2. Materials and Methods

### 2.1. Glasshouse Experiment

In May 2023, a greenhouse experiment was conducted using 20 sweet pepper (Solanaceae) seedlings of the ‘Quadrato d’asti giallo’ variety from “Eurogarden d.o.o.” Each seedling was planted in a 3 L pot filled with Bio Plantella Universal soil. The greenhouse at the Biotechnical Faculty in Ljubljana, Slovenia, maintained daytime temperatures of 21 °C (RH = 55%) and night-time temperatures of 11 °C (RH = 75%), with plants watered every four days. Fifty wireworms were collected from the Biotechnical Faculty grounds (46°04′ N, 14°31′ E, 299 m a.s.l.) and identified as *A. lineatus* by their abdominal raster pattern [25]. Ten pots were infested with five wireworms each, while the other ten pots remained uninfested. The experiment ended in June 2023 with the collection of leaf (20 g/replication) and root samples (10 g/replication).

### 2.2. Determination of Antioxidants and Photosynthetic Pigments

Sampling occurred on a clear day between 11 a.m. and 2 p.m. Leaves were defoliated and immediately frozen in dry ice. Roots were carefully sampled and washed with warm water, dried with a towel, then frozen with dry ice, and stored at −80 °C. The plant material was freeze-dried and ground as per Tausz et al. [26].

For the determination of thiols (total cysteine and total GSH), 40 mg of lyophilized plant material was extracted with 2 mL of 0.1 M HCl, containing 60 mg of polyvinylpolypyrrolidone to remove phenolics. After homogenization and centrifugation, 280 μL of the extract was mixed with 420 μL of CHES buffer and 70 μL of dithiothreitol for 1 h. The sample was then labeled with 50 μL of monobromobimane in the dark for 15 min and the reaction was stopped with 600 μL of methanesulfonic acid.

For oxidized thiols, 30 μL of N-ethylmaleimide and 600 μL of CHES buffer were added to 400 μL of the thiol extract, incubated for 15 min, and excess N-ethylmaleimide was removed by toluene extraction. The remaining oxidized thiols were reduced and derivatized with monobromobimane in the dark at RT for 15 min. Derivatization was stopped by adding a 600 μL aliquot of 0.25% (*v*/*v*) methanesulfonic acid. Prepared samples were analyzed using gradient high-pressure liquid chromatography system (HPLC): Waters 2695 HPLC system, Waters 2475 Multi Fluorescence detector (excitation: 380 nm wavelength; emission: 480 nm wavelength), column: Grace Waters Spheresorb ODS–2.5 μm, 250 × 4.6 mm, column temperature 25 °C, sample temperature 4 °C. Solvent A: double distilled water containing 5% methanol and 0.25% acetic acid, pH 3.9; Solvent B: 90% methanol containing 0.22% acetic acid in water, pH 3.9. Gradient: 20 min 90% of solvent A and 10% of solvent B, 20–30 min 85% of solvent A and 15% of solvent B, 30–35 min 5% solvent A and 95% solvent B, 35 min, 90% of solvent A and 10% of solvent B. Run time: 40 min with flow rate 1 mL/min. For standard solutions, reduced glutathione (GSH, M = 307.3 g/mol) and cysteine (M = 121.16 g/mol) were used, which was obtained from Sigma–Aldrich (St. Louis, MO, USA).

Total ascorbate levels were measured using a modified reversed-phase HPLC method. A sample of 30 mg of lyophilized plant material was extracted with 3 mL of 3% metaphosphoric acid, with PVP added to remove phenolics. Extracts were analyzed directly or after derivatization. For total ascorbate, 600 μL of extract was mixed with 280 μL 0.4 M Tris buffer and then 50 μL 0.26 M dithiothreitol was added for the reduction of oxidized form. After 10 min incubation at room temperature in the dark, 100 μL of 8.5% orthophosphoric acid was added to stop the reaction. For reduced ascorbate, the extract was processed similarly by adding aliquote of distilled water instead of ditihiothreitol. Analyses of total ascorbate were conducted by using an isocratic HPLC method on Waters 2695 HPLC system and Waters 996 PDA detector (excitation: 245 nm). Column: Phenomenex, Synergi 4 μm Hydro–RP 80 A, 150 × 4.60 mm. Solvent: 32.5 mM NaH2PO4 (pH 2.2). Run time: 20 min, flow rate 0.5 mL/min. For standard solutions, ascorbic acid (M = 176.13 g/mol, Sigma-Aldrich, St. Louis, MO, USA) was used. Photosynthetic pigments were analyzed using gradient HPLC method on Waters 2695 HPLC system and Waters 2475 Multi Fluorescence Detector (excitation: 440 nm). Column Spherisorb S5 ODS2 25 × 4.6 μm. Mobile phase: (A) acetonitrile:water:methanol (100:10:5); (B) acetone:ethylacetate (2:1 *v*/*v*); Gradient: 10% (B) to 80% (B) in 17 min, hold 5 min, return in 5 min. Flow rate 1 mL min^–1^.

### 2.3. Pepper Roots’ Volatile Organic Compounds Analyses

To prepare sweet pepper roots for analysis, they were flash-frozen in liquid nitrogen and ground with a ceramic mortar and pestle. About 0.5 g of the sample was placed in a 20 mL headspace vial, sealed, and incubated at 50 °C for 30 min, based on observed differences in volatile profiles between control and wireworm-attacked plants. A solid-phase microextraction (SPME) fiber coated with 100 μm polydimethylsiloxane (PDMS) thick film was exposed to the vial’s headspace and preconditioned in the GC injection port for 10 min before sampling. The SPME sampling technique was chosen due to its known ability to preconcentrate volatile analytes. PDMS coating is a general purpose sorbent known for its ability to absorb a wide range of VOCs, which has been proven to be the best choice in our similar recent research work [27].

SPME–GC-MS analysis was performed using an Agilent 7890B gas chromatograph coupled with an Agilent 5977B mass spectrometer. The column used was a ZB-5HT-Inferno (5% diphenyl-95% dimethylsiloxane; 20 m × 0.18 mm i.d. with 0.18 μm film thickness). The oven temperature program started at 30 °C (5 min), ramped at 12 °C/min to 250 °C (2 min). The injector was in splitless mode at 250 °C, with an initial pressure surge of 200 kPa for 1.5 min and a 1 mm i.d. glass liner. Helium was used as the carrier gas at 0.6 mL/min, and the transfer line was set to 260 °C. The mass spectrometer operated at 230 °C with 70 eV ionization energy, scanning from 46 to 350 amu at 5 scans/s. The analytes were identified using the provided NIST MS search (version 2.3.) database.

### 2.4. Source and Maintenance of Parasitic Nematodes and Synthetic Volatile Organic Compounds

For the chemotaxis assay, we used three commercially available EPN species—*S. feltiae*, *S. carpocapsae*, and *H. bacteriophora*—sourced from Koppert B.V. (Berkel en Rodenrijs, The Netherlands). These nematodes were reared on final-instar larvae of the wax moth (*Galleria mellonella* [L.], Lepidoptera: Pyralidae) and stored at 2000 IJs/mL at 4 °C. Only nematodes less than two weeks old were used, with their concentration determined as per Laznik and Trdan [5]. Nematode viability was checked before testing.

We collected *Arion vulgaris* Moquin-Tandon specimens from Ljubljana, near the river, Glinščica, Slovenia (46°05′ N, 14°33′ E), between July and September 2021. Species identification was conducted using identification charts [27], and collected slugs (n = 100) were rinsed with 0.9% saline solution following the protocol by Pieterse et al. [23]. These nematodes were cultured in freeze-killed slugs and extracted after 10 days using methods similar to those for EPNs [24]. Nematodes were stored in M9 buffer at 4 °C and used if they were less than two weeks old and had over 95% viability.

Two potential chemotaxis compounds were selected for the chemotaxis assay based on analyses of sweet pepper roots and leaves. The first was VOC hexanal (PubChem CID: 6184) and the second was the amino acid root exudate cysteine (PubChem CID: 5862). The synthetic compounds were tested at a concentration of 0.03 μg/mL, representing typical levels in the rhizosphere [27,28,29]. We used 96% ethanol as the solvent for hexanal and distilled water for cysteine.

### 2.5. Chemotaxis Assay

**Figure 1:**
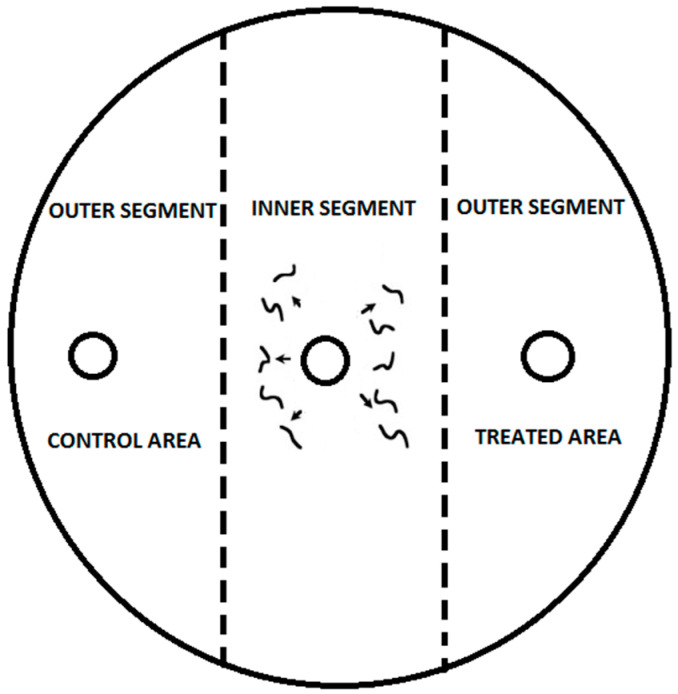
To start the experiment, three 1 cm diameter marks were made on the bottom of each Petri dish: one in the center and two 1.5 cm from the edge on the right and left. Then, 0.03 μg/mL tested substance was applied to the right side of the agar, while the left side was treated with 10 μL of distilled water/96% ethanol as the control. VOCs were applied just before introducing 100 IJs of nematodes, in a 50 μL droplet, to the center of the agar. The control setup also used distilled water/96% ethanol for both sides and placed a 50 μL droplet of 100 IJs in the center.

For the chemotaxis assay, Petri dishes (9 cm diameter) were filled with 25 mL of 1.6% technical agar (Biolife, Milan, Italy) and supplemented with 5 mM potassium phosphate (pH 6.0), 1 mM CaCl_2_, and 1 mM MgSO_4_. Each treatment was replicated twenty times and kept in a rearing chamber (RK-900 CH, Kambič Laboratory equipment, Semič, Slovenia) at 18 or 20 °C with 75% RH. After 24 h, nematodes were frozen at −20 °C for 3 min to immobilize them. Nematodes were counted using a Nikon C-PS binocular microscope at 25× magnification. The chemotaxis index (CI) was calculated using the method adapted from Bargmann and Horvitz [30] and Laznik and Trdan [5,29] (Figure 1).

CI = (% of IJs in the treatment area − % of IJs in the control area)/100%(1)

The CI ranges from 1.0 (indicating perfect attraction) to −1.0 (suggesting perfect repulsion). In the experiments described herein, compounds were categorized as follows: ≥0.2 as an attractant, from 0.2 to 0.1 as a weak attractant, from 0.1 to −0.1 as having no effect, from −0.1 to −0.2 as a weak repellent, and ≤−0.2 as a repellent to EPNs [5,29].

### 2.6. Statistical Analysis

#### 2.6.1. Biochemical Analyses of Photosynthetic Pigments and Antioxidants

The mean and standard deviation of the antioxidants’ and photosynthetic pigments’ biochemical data associated with wireworm infestation underwent analysis through independent sample *t*-test, with significant differences denoted by asterisks (*p* < 0.05). Statistical computations were conducted utilizing IBM SPSS Statistics 21 (New York, NY, USA, 2012). Graphs are represented with the help of OriginPro 2024 program (OriginLab Corporation, Northampton, MA, USA).

#### 2.6.2. Chemotaxis Assay

In the chemotaxis assay, a paired Student *t*-test was used to assess nematode migration from the center to the outer segments of the Petri dish, with significance set at *p* < 0.05. One-way ANOVA compared the movement of IJs to the outer versus inner segments across different treatments, considering VOCs, nematode species, temperature, and replication. Significant interactions were found only between VOCs, nematode species, and temperature. Additional one-way ANOVA analyzed CI values for different nematode species’ responses to VOCs, with means compared using Duncan’s multiple range test at *p* < 0.05. Data are presented as mean ± S.E. Statistical analyses were performed with Statgraphics Plus for Windows 4.0, and figures were created using MS Office Excel 2010.

## 3. Results

### 3.1. Antioxidant Concentrations and Redox State

**Figure 2:**
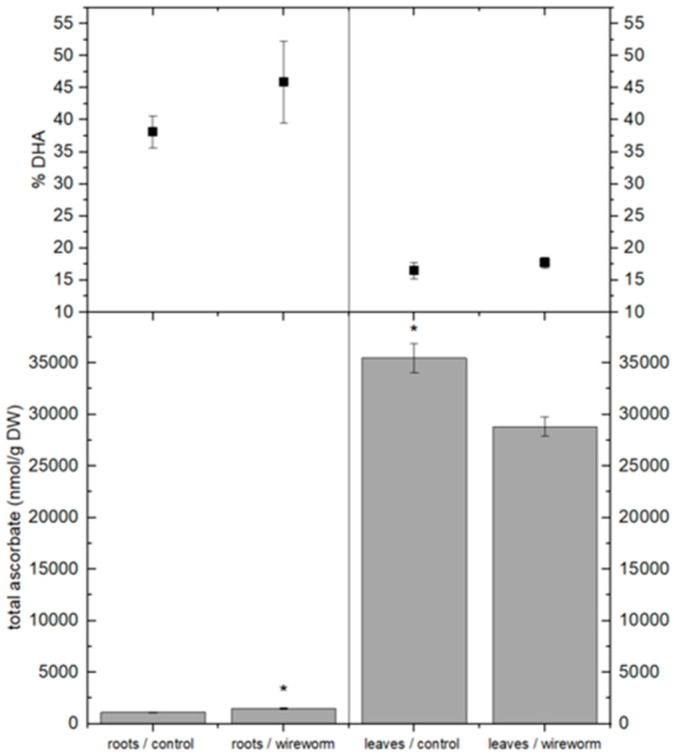
Concentrations of total ascorbate (nmol/g DW) and dehydroascorbate (% of total) in the roots and leaves of non-attacked *C. annuum* plants and *C. annuum* plants attacked by *A. lineatus*. Asterisks * indicate statistical differences (*p* < 0.05) tested with an independent *t*-test between roots and leaves from attacked and non-attacked plants.

The ascorbic acid content in the leaves of wireworm-infested sweet pepper plants was 19% lower compared to the control plants, both in its reduced and total forms. However, this infestation did not alter the redox state. In contrast, the roots of the infested plants showed increases of 26% and 29% in reduced and total ascorbate levels, respectively. Additionally, there was a notable increase in the percentage of dehydroascorbic acid (% DHA) (Figure 2).

**Figure 3:**
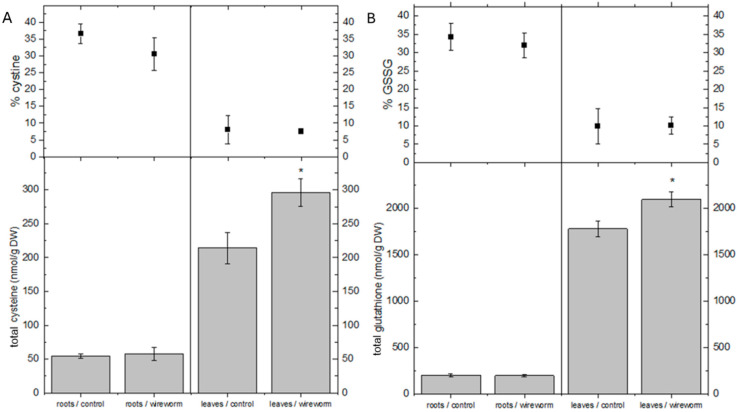
(**A**) Concentrations of cysteine (nmol/g DW) and cystine (% of total) and (**B**) total glutathione and GSSG (% of total) in the roots and leaves of non-attacked *C. annuum* plants and *C. annuum* plants attacked by *A. lineatus*. Asterisks * indicate statistical differences (*p* < 0.05) tested with an independent sample *t*-test between roots and leaves from attacked and non-attacked plants.

The leaves of infested plants exhibited significantly higher total cysteine and glutathione contents, increasing by 38% and 11%, respectively, compared to control plants. No significant changes in the contents and the redox state of both thiols were observed in the roots (Figure 3A,B).

### 3.2. Photosynthetic Pigment Concentrations

The pigment analysis revealed significant increases in total carotenoids and chlorophylls in wireworm-infested plants. Specifically, neoxanthin and lutein levels were higher by 22% and 18%, respectively. There was also a significant increase in the pigments of the xanthophyll cycle. The ratio of violaxanthin/antheraxanthin+zeaxanthin (V/A+Z) significantly decreased, indicating a shift towards a more de-epoxidized state. Furthermore, the β-carotene levels increased by 23% (Table 1).

Additionally, we observed wireworm-induced changes in the chlorophyll contents. The chlorophyll a and chlorophyll b levels increased significantly in the infested leaves by 14% and 16%, respectively. However, the chlorophyll a-to-chlorophyll b ratio remained unchanged. The ratios of carotenoids to chlorophylls, specifically VAZ/chl a+b and β-carotene/chl a+b, also increased (Table 1).

### 3.3. Sweet Pepper Root Volatile Organic Compound Analyses

The analysis of volatile components in pepper roots showed the presence of various compounds from different chemical classes, with changes in their levels observed in the infected roots compared to the control group depending on the compound or its class. Specifically, pepper plants attacked by wireworms exhibited a decrease in the levels of certain VOCs, including limonene (by about −47%), methyl salicylate, and benzyl salicylate compared to the control group. On the other hand, the roots or aerial parts of the attacked plants showed increased levels of several VOCs, among them, most notably, hexanal (by about +43%), neoisopulegol, nonanal, phenylethyl alcohol, m-di-tert-butylbenzene, and trans-β-ionone (Table 2). It should be noted that the observed relative level increases or decreases were very variable among the majority of detected compounds, but the trends (i.e., increase or decrease) were consistent. The most consistent level changes, however, were observed for the most volatile compounds (i.e., hexanal and limonene).

### 3.4. Nematode Chemoattraction towards VOCs

#### 3.4.1. Nematode Motility

**Figure 4:**
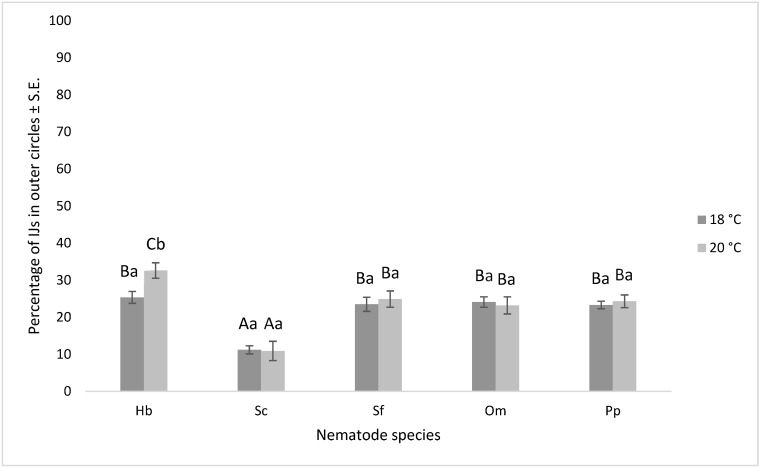
The chart displays the percentage of different nematode IJs (infective juveniles) in the outer circles after 24 h, depending on the temperature used in the experiment. The error bars represent the standard error. Capital letters denote statistically significant differences (*p* < 0.05) among the various nematode species at the same temperature, while lowercase letters indicate statistically significant differences (*p* < 0.05) among the different temperatures within the same nematode species. The nematode species used in the experiment are as follows: Hb = *Heterorhabditis bacteriophora*; Sc = *Steinernema carpocapsae*; Sf = *Steinernema feltiae*; Om = *Oscheius myriophilus*; Pp = *Phasmarhabditis papillosa*.

The mobility of nematode infective juveniles (IJs) within an assay dish, from inner to outer areas, is referred to as their motility. Our investigation revealed that nematode species, chemotaxis compounds, temperature, and their interactions significantly influenced nematode motility, as indicated in Table 3. Notably, *H. bacteriophora* (29.0 ± 1.2%) exhibited significantly higher proportions of IJs in the outer circles compared to *S. carpocapsae* (11.1 ± 1.0%). Temperature emerged as a significant factor for *H. bacteriophora*, while other nematode species tested were unaffected by temperature shifts (as depicted in Figure 4). Among the chemoattractants tested, cysteine demonstrated the most pronounced stimulation of nematode movement from inner to outer areas of the assay dish.

#### 3.4.2. Chemotaxis Index

The movement preference of nematode IJs was assessed using CI. The ANOVA results indicated that various factors and their interactions influenced the CI values (Table 4). At 18 and 20 °C, *H. bacteriophora*, *S. feltiae*, *O. myriophilus*, and *P. papillosa* nematodes showed preferential movement towards the outer circles when hexanal was used as a weak attractant (Table 5). However, *S. carpocapsae* showed no behavioral response to hexanal at 18 and 20 °C. Cysteine attracted *H. bacteriophora* (CI = 0.26 ± 0.02) and acted as a weak attractant for the *S. feltiae* nematode. On the other hand, it had no effect on the preferential movement of the *S. carpocapsae* nematode (Table 5).

## 4. Discussion

Research into underground multitrophic communication—interactions among plant roots, insects, and parasitic nematodes—is relatively new. Pioneering work by Rasmann et al. [1] demonstrated that maize roots emit specific volatile organic compounds (VOCs) when damaged, which affect the movement of the EPN *H. megidis*. This discovery has spurred the development of the field of underground multitrophic communication [2,5,27]. However, studies on how EPNs respond to VOCs from plant roots, whether damaged or not, are limited, and the role of antioxidants in belowground plant responses to biotic stress is not well understood [8,13,14,15]. Also, a (semi)quantitative analysis of VOCs in such complex samples such as whole plant tissue represents several challenges which can be hardly addressed using SPME sampling. Nonetheless, a qualitative SPME-GC-MS analysis is still able to provide valuable data regarding the identity of semiochemicals, while at the same time, also a means for analyte preconcentration. Our study investigated how wireworm infestation affects the ascorbate–glutathione system in sweet pepper plants, focusing on its impact on photoprotective defenses and chemotaxis. We found that wireworm infestation significantly decreased the total ascorbate in the leaves, but increased both the reduced and total ascorbate in the roots. This contrasts with our previous research on lettuce [27], where both roots and leaves showed higher ascorbate levels. Additionally, infested sweet pepper leaves had elevated total cysteine and glutathione levels, while the redox state of these thiols in the roots remained unchanged.

In our previous study on wireworm-infested lettuce [27], we observed significantly higher levels of GSSG and a trend toward increased total glutathione in both roots and leaves compared to controls. Similar results were reported in other research [31,32,33], where feeding sites of biting insects showed elevated glutathione levels. Additionally, changes in glutathione peroxidase and transferase activities were noted in the leaves of *Arabidopsis thaliana* infested by mites [31]. Other studies found increased ascorbate and glutathione levels in both roots and leaves during nematode attacks [34]. The role of antioxidant defense mechanisms in mediating communication between aboveground and belowground plant parts remains unclear, particularly regarding their spatial and temporal dynamics. Our findings, which include decreased ascorbate in leaves and increased levels in roots alongside elevated glutathione in both tissues, suggest that roots act as a sink for ascorbate–glutathione redistribution from leaves. Some researchers propose that reduced ascorbate levels combined with a high glutathione content triggers defense responses by activating defense genes [32,33,34].

A pigment analysis revealed significant increases in the total carotenoids and chlorophylls in wireworm-infested plants. There was also a notable rise in xanthophyll cycle pigments, with a significant decrease in the violaxanthin/antheraxanthin+zeaxanthin (V/A+Z) ratio, indicating a shift to a more de-epoxidized state [35]. This shift is essential for dissipating excess energy and protecting photosynthetic membranes. The increased de-epoxidation and total xanthophyll levels suggest the enhanced protection of thylakoid membrane proteins. These results are consistent with previous findings of increased zeaxanthin and decreased violaxanthin after wireworm attacks [27]. Additionally, increases in other carotenoids like β-carotene, neoxanthin, and lutein, which are important chloroplast antioxidants in oxidative stress responses [36,37,38,39,40], were observed.

Plant damage can significantly alter the quantity and quality of root exudates [1]. Detecting these changes is challenging due to the variety of compounds involved, making mass spectrometry crucial for identifying key shifts. One notable compound, hexanal, increases in sweet pepper root secretions when plants are infested by wireworms. Produced through the oxidation of fatty acids like linoleic acid by enzymes such as lipoxygenases, hexanal contributes to the “green” smell of freshly cut grass and is found in many fruits and vegetables [41,42]. It also plays a role in plant defense, acting as a signaling molecule that helps trigger defense responses, including attracting natural predators and deterring pests [43]. Our study’s findings align with these established observations. Although the trend of elevated cysteine levels in the roots of wireworm-infested plants was not significant, we chose cysteine for the chemotaxis assay because it is known to have a chemoattractant effect on the bacterial community of the rhizosphere as a root exudate [43]. In addition, we chose cysteine as a direct precursor of glutathione based on the suggestion that roots serve as a sink for the redistribution of ascorbate and glutathione from leaves, based on our results, showing a decrease in ascorbate in leaves and an increase in roots with concomitant increases in thiols in leaves.

Our study revealed that nematode infective juveniles (IJs) respond differently to tested chemotaxis compound, influenced by factors like a specific compound, nematode species, and environmental conditions such as temperature. These findings are in line with our previous research on nematode responses to insect-damaged carrot and lettuce roots [5,27]. We tested compounds like hexanal and cysteine, which are released by damaged sweet pepper roots. While these compounds generally attracted most nematodes, *S. carpocapsae* did not show the same attraction, emphasizing the importance of root exudates in nematode behavior and navigation.

The differences in chemosensory responses among nematode species relate to their foraging strategies [44,45,46,47]. Cruisers like *H. bacteriophora* actively seek hosts and are more responsive to VOCs indicating potential hosts. In contrast, ambushers like *S. carpocapsae* wait for hosts to come to them [44,45,46,47], which explains their lack of attraction to hexanal and cysteine in our study. The chemosensory behaviors of *P. papillosa* and *O. myriophilus* are less understood. Although previous research [45,46] has examined *Phasmarhabditis* responses to host mucus, these findings are not directly comparable to ours.

Our study showed lower chemotaxis index (CI) values compared to similar research [2,5], with the highest CI of 0.26 ± 0.02 observed for *H. bacteriophora* IJs exposed to cysteine. Previous studies [2,27] reported CI values above ±0.5 for *Heterorhabditis* and *Steinernema* species. These differences may be due to variations in the methodology or strain-specific traits of the nematodes, as noted in earlier research [2,44,45]. Additionally, laboratory conditions might not fully replicate the nematodes’ natural environment [47].

Temperature significantly influenced nematode movement in response to chemotaxis compounds, with increased motility at 20 °C compared to lower temperatures. Although previous studies [48,49] suggested that *Steinernema* species are more active at lower temperatures than *Heterorhabditis*, our results did not support this. Differences may be due to nematode strains adapting to laboratory conditions, which might not reflect their natural thermal preferences [48,49], or because chemotaxis compound emissions may be more pronounced at higher temperatures [50].

## 5. Conclusions

In conclusion, our study highlights the complex interactions between plant defense mechanisms and nematode behavior in the multitrophic context of wireworms, sweet pepper plants, and parasitic nematodes. We observed significant changes in the antioxidant systems of wireworm-infested plants, including shifts in ascorbate and glutathione levels. Specifically, ascorbate decreased in leaves but increased in roots, while glutathione levels rose in both tissues. These findings suggest that roots act as a sink for the ascorbate–glutathione system, indicating that the redistribution may bolster root defense.

Our findings also emphasize the role of root exudates like hexanal and cysteine in influencing the nematode behavior. Despite variability in the chemotactic responses among nematode species, root exudates were shown to impact nematode movement and host-seeking behavior. The differences in the chemotaxis indices and the effects of temperature highlight the importance of considering these factors in biological control strategies.

This research provides valuable insights into underground multitrophic communication and suggests that effective nematode management will require understanding species-specific responses and environmental conditions. Further research is needed to fully explore plant–nematode interactions and improve biological control methods in agriculture.

## Figures and Tables

**Table 1:** Concentrations of pigments (mg/g DW) together with the percentage increase/decrease in the leaves of non-attacked *C. annuum* plants and *C. annuum* plants attacked by *A. lineatus* plants. Asterisks * indicate statistical differences (*p* < 0.05) tested with an independent sample *t*-test between roots and leaves from attacked and non-attacked plants.

Pigment Concentrations (mg/g DW)	Leaves Control	Leaves Wireworm	Sig.	% Increase/Decrease
**Neoxanthin**	291.4 ± 46.4	266.6 ± 57.3	*	−9
**Violaxanthin**	130.5 ± 36.2	148.5 ± 30.9		14
**Antheraxanthin**	31.4 ± 12.1	34.6 ± 10.9		10
**Zeaxanthin**	111.7 ± 8.1	157.3 ± 7.1	*	41
**VAZ**	273.6 ± 56.4	340.3 ± 49.0	*	24
**V/A+Z**	0.9 ± 1.8	0.8 ± 1.7	*	−15
**Lutein**	650.4 ± 146.7	769.7 ± 172.9	*	18
**Total carotenoids**	1215.4 ± 49.9	1376.6 ± 55.9		13
**Chl a**	3865.9 ± 336.4	4374.8 ± 350.3	*	13
**Chl b**	1671.6 ± 862.4	1936.6 ± 350.3	*	16
**ß-carotene**	435.2 ± 106.8	537.6 ± 82.1	*	24
**Chl a+b**	5537.5 ± 1139.9	6311.4 ± 1280.4	*	14
**Chl a/b**	2.3 ± 0.1	2.25 ± 0.2		−2
**VAZ/chl a+b**	0.049 ± 0.005	0.054 ± 0.004		9
**β-carotene/chl a+b**	0.079 ± 0.001	0.085 ± 0.002	*	8

**Table 2:** Results of SPME-GC-MS analysis of volatile organic compounds (VOC) emitted by aerial parts (AP) and roots (R) of *C. annuum* plants non-attacked (C-control) and attacked by *A. lineatus* wireworms (W).

VOC	CAS Number	Occurrence	C	W
**hexanal**	66-25-1	R	+	++
**2-hexenal**	6728-26-3	AP, R	+	++
**limonene**	5989-27-5	R	++	+
**neoisopulegol**	21290-09-5	R	+	++
**nonanal**	124-19-6	AP	+	++
**phenylethyl alcohol**	60-12-8	R	+	++
**methyl salicylate**	119-36-8	AP, R	++	+
**m-di-tert-butylbenzene**	1014-60-4	AP, R	+	++
**trans-β-ionone**	79-77-6	AP	+	++
**3-methyl-1,2-cyclopentanediol**	27583-37-5	AP	+	++
**benzyl salicylate**	118-58-1	AP	++	+
**tetradecanal**	124-25-4	R	+	++
**pentadecanal**	2765-11-9	AP, R	+	++
**18-norabietane**	2221-95-6	R	+	++
**octadecanal**	638-66-4	R	+	++
**octacosane**	630-02-4	R	+	++
**dibutyl phthalate**	84-74-2	R	−	+

+ = VOC is present; − = VOC is not present; ++ = level of VOC is increased compared to the other group.

**Table 3:** ANOVA results for the directional movement of infective juveniles (IJs) from the inner to the outer segments of the Petri dish.

Factor	Sum of Squares	Df	F	*p*
Species (S)	21,450.1	4	75.41	<0.001
VOCs (V)	2977.84	3	11.44	<0.001
Temperature (T)	427.888	1	6.02	0.0145
Replication (R)	4804.49	19	3.56	<0.001
S x V	18,621.07	12	18.22	<0.001
S x T	1268.77	4	4.46	0.0015
V x T	1605.9	3	5.03	0.0022
S x V x T	2785.02	12	2.57	0.0026
Residual	39,183.1	551		
Total (Corrected)	93,124.178	599		

**Table 4:** ANOVA results for the chemotaxis index values.

Factor	Sum of Squares	Df	F	*p*
Species (S)	0.73	4	73.03	<0.01
VOCs (V)	3.04	3	402.21	<0.01
Temperature (T)	0.11	1	43.12	<0.01
Replication (R)	0.11	19	2.34	0.0011
S x V	0.40	12	14.24	<0.01
S x T	0.09	4	8.55	<0.01
V x T	0.02	3	2.41	0.0673
S x V x T	0.17	12	6.05	<0.01
Residual	1.39	551		
Total (Corrected)	6.06	599		

**Table 5:** Effect of different volatile organic compounds on the chemotactic response of the nematode species after 24 h. Data represent the mean value of the chemotaxis index ± standard error. Statistically significant (*p* < 0.05) differences between nematodes treated with the same volatile organic compound and the same temperature are denoted with different letters. The nematode species used in the experiment are as follows: Hb = *Heterorhabditis bacteriophora*; Sc = *Steinernema carpocapsae*; Sf = *Steinernema feltiae*; Om = *Oscheius myriophilus*; Pp = *Phasmarhabditis papillosa*. The chemotaxis index values are categorized as follows: values ≥ 0.2 are considered as an attractant, values between 0.2 and 0.1 are classified as a weak attractant, values between 0.1 and −0.1 have no effect, values between −0.1 and −0.2 are categorized as a weak repellent, and values ≤ −0.2 are considered as a repellent to nematodes.

A	18 °C				
	Hb	Sc	Sf	Om	Pp
**Hexanal**	0.17 ± 0.01 d	0.03 ± 0.00 a	0.14 ± 0.00 c	0.11 ± 0.01 b	0.10 ± 0.00 c
**Cysteine**	0.11 ± 0.01 b	0.05 ± 0.01 a	0.18 ± 0.01 d	0.11 ± 0.01 b	0.14 ± 0.01 d
**Control (DW)**	−0.02 ± 0.00 a	−0.03 ± 0.01 a	−0.03 ± 0.01 a	−0.02 ± 0.00 a	−0.03 ± 0.01 a
**Control (E)**	−0.01 ± 0.01 a	−0.02 ± 0.00 a	−0.02 ± 0.01 a	−0.01 ± 0.01 a	0.01 ± 0.01 b
**B**	**20 °C**				
	**Hb**	**Sc**	**Sf**	**Om**	**Pp**
**Hexanal**	0.19 ± 0.02 c	0.04 ± 0.00 a	0.19 ± 0.02 c	0.12 ± 0.01 b	0.18 ± 0.01 c
**Cysteine**	0.26 ± 0.02 e	0.05 ± 0.01 a	0.17 ± 0.01 c	0.07 ± 0.00 b	0.19 ± 0.00 d
**Control**	−0.01 ± 0.00 a	−0.01 ± 0.00 a	−0.02 ± 0.01 a	−0.01 ± 0.00 a	−0.02 ± 0.01 a
**Control (E)**	0.00 ± 0.01 a	−0.01 ± 0.01 a	−0.01 ± 0.01 a	0.00 ± 0.01 a	0.01 ± 0.01 b

## Data Availability

The data presented in this study are available on request from the corresponding author.
